# Editorial: The rights and needs of children during times of war and conflict

**DOI:** 10.3389/fpsyg.2026.1817650

**Published:** 2026-03-17

**Authors:** Tali Gal, Heba F. Zedan, Sharon Bessell

**Affiliations:** 1 The Minerva Center for Human Rights, Hebrew University of Jerusalem, Jerusalem, Israel; 2 Department of Policy & Governance, The Crawford School of Public Policy, Australian National University, Canberra, ACT, Australia

**Keywords:** armed conflict, children's needs, children's rights, convention on the rights of the child, research topic, war

Recent years have seen unprecedented levels of harm to children in armed conflict. The UN verified 41,370 grave violations in 2024, the highest in the mandate's history, and UNICEF estimates 473 million children (about one in six) now live in conflict-affected areas. Armed conflicts are producing extreme violations of children's rights in staggering numbers across many settings, including the Gaza Strip, Sudan, Ukraine, the Democratic Republic of the Congo, Somalia, Nigeria, Myanmar, Haiti, Lebanon, Israel and the occupied Palestinian Territory, Mozambique, and Ethiopia.

Wars are the product of decisions made by political leaders, yet their most profound and enduring consequences fall on those with the least power to initiate, shape, or end them: children. Across settings, children experience overlapping harms—physical injury and death, family separation and displacement, hunger and poverty, disrupted education and development, and exposure to chronic fear and violence. Too often, they are also excluded from peacebuilding processes that will determine their futures.

Since the Geneva Declaration of the Rights of the Child of 1924 (1924), the international community has repeatedly affirmed that children require special protection in war. This commitment was later codified in binding form through the Convention on the Rights of the Child (1989), which recognizes children's rights to survival, development, protection, and participation, and obligates states to safeguard children affected by armed conflict. Yet contemporary conflicts demonstrate a persistent gap between legal norms and children's lived realities. The widespread harm to children across current wars is therefore a humanitarian crisis and a failure of implementation of well-established international obligations.

Despite these instruments, children's rights are invoked in contemporary wars more often than they are realized. An extensive legal framework and moral consensus has failed to prevent children being displaced and separated from family, exposed to chronic insecurity, denied education and health care, and subjected to violations that shape development across generations. This Research Topic treats the gap between promise and practice as both empirical and normative, asking not only what war does to children, but how children and caregivers navigate rights in motion under constraint, surveillance, and conditions where ordinary life becomes high-risk.

The initiative to create this Research Topic emerged while two of us (T.G., H.Z.), as Israeli citizens, were directly impacted by the Israel–Gaza war and lived under recurrent warnings of rocket and missile fire from multiple fronts. Yet even as we worried for our families' safety and emotional stability, it was the scale of violations of children's rights in Gaza that ultimately motivated us to edit this Research Topic. With estimates now suggesting that over 70,000 people have been killed in Gaza, the war ranks among the deadliest conflicts of the past decade in terms of civilian deaths over a compressed timeframe, even as other recent wars have produced larger overall death tolls across larger populations and longer periods. Particularly devastating are the reported deaths of children and adolescents in Gaza between October 2023 and October 2025: the Gaza Ministry of Health's casualty records and published lists indicate that more than 20,000 of those killed were minors, with especially harrowing losses among the very young. One of us (T.G.), as a Jewish Israeli, has participated in protests and other forms of activism against the Israeli government and against the war, and has felt deep frustration, anger, and shame at Israel's actions, alongside grief for the heavy toll borne by Israeli children and for the harrowing trauma of the October 7 attack. H.Z., a Palestinian scholar from Israel, approached the topic from lived proximity to conflict and from experiencing limited freedom of speech during the war. These conditions reinforced the importance of careful, evidence-based engagement with children's experiences. Editing this Research Topic became a way to sustain attention to children's rights and wellbeing within an academic framework. SB, a scholar of children's human rights, lives in a country free from armed conflict, but with a history of deep violence against Aboriginal and Torres Strait Islander peoples. Editing this volume was a means of illuminating both the wrongs committed against children during war and standing with scholars seeking to illuminate rights violations in the most difficult of circumstances. For all of us, a Research Topic dedicated to the harms of war and armed conflict on children came to feel like the most meaningful form of political action available to us as scholars committed to the human rights of all children. We hope this Research Topic will not only contribute to global knowledge about children's rights and needs in war; it will also support activists and communities working to promote peace, stability, and safety for children worldwide.

The 14 contributions in this Research Topic examine the complex experiences, wellbeing, and human rights of children affected by war. Together, they trace children's lives across acute crises and protracted emergencies, and they identify relational, institutional, legal, and ethical leverage points through which protection, provision, and participation might be strengthened.

The interdisciplinary nature of the papers helps capture the interconnected challenges children face across contexts shaped by violence and displacement. Across geographic and political settings, the Research Topic shares a commitment to child-focused analysis that takes children's rights, needs, and perspectives seriously.

We organize the contributions in this Research Topic according to five thematic frameworks emerging in the context of wars and armed conflict:

(1) Systemic failure of the ecosystems of children's lives: Three papers foreground how war dismantles the conditions that make childhood possible. Bessell and O'Sullivan offer a rights-based review of the failure to protect children in Gaza and the destruction of the socio-ecological systems that sustain children. Zedan documents continuous traumatic stress among Palestinian adolescents in East Jerusalem, emphasizing chronic insecurity, mistrust in protective systems, and the embodied transmission of fear. And Nasser analyzes Palestinian Nakba narratives to show how displacement and settler-colonial violence can “unchild” children, while also illuminating memory and childhood as sites of survival and resistance.

(2) Caretakers' mental health as a buffer in acute situations: This section centers the family as the first protective system in crisis. Fennig et al. provide rare qualitative evidence on children's and caregivers' early psychological responses immediately after release from war captivity, and the immediate, developmentally tailored interventions used by practitioners. Enav and Mayer show that parental reflective functioning can mitigate the pathway from parental war exposure to child distress among displaced families with young children. And Buchnik-Atzil et al. similarly find that maternal self-efficacy and parent–child communication buffer the association between maternal anxiety and young children's emotional distress during wartime.

(3) Threats to family support in the context of refuge: These contributions examine how displacement and life in refugee camps reshape caregiving, decision-making, and children's daily rights across time and geography. Klassen et al. trace how Syrian refugee mothers' practices of child supervision shift across stages of conflict, flight, transit, and resettlement, highlighting tensions between provision, protection, and participation. Presler-Marshall et al. analyze child marriage decision-making among refugees in Jordan, showing how legal and economic precarity and gendered obligations constrain choices for girls and families. Akesson et al. focus on Rohingya refugee camps, detailing how chronic deprivation, insecurity, and restricted opportunities undermine caregivers' capacity to meet children's needs across the perinatal and early childhood period.

(4) Children's agency in peacebuilding: Two papers insist that children are not only subjects of protection but also agents of recovery and peace. Glos et al. show how play supports psychological recovery, local peacebuilding, and reintegration while advancing children's right to play among child refugees and migrants in Chile. Abidi foregrounds the peace perspectives of children and youth affected by armed violence, highlighting their emphasis on inclusion, interpersonal peace, and peacebuilding skills, and offering recommendations for meaningful child engagement in peace and security agendas.

(5) Law, rights discourse, and ethics in war: This section addresses the normative and methodological infrastructure needed to respond to war's harms. Boothby reviews legal frameworks and the persistent practical failures to prevent family separation and achieve reunification in armed conflicts. von Denkowski confronts the ethical challenges of research with conflict-affected children and argues for a reflexive, care-ethical approach to “ethically important moments” in practice. And Rum et al. integrate a CRC-based normative framework with adolescents' own reports from wartime Israel, illuminating how protection, provision, and participation are experienced and unequally distributed during prolonged conflict.

Taken together, these contributions offer a multidimensional understanding of the needs, rights, harms, and sources of protection and resilience of children and adolescents in contexts of war and armed conflict. They show, on the one hand, how war jeopardizes the full spectrum of children's rights and basic needs, and on the other, the central role of caretakers, community members, and public actors in protecting and promoting children's safety, wellbeing, and human rights.
